# Implications of Potential US FDA Approval of Psychedelics or Psychedelic-Adjacents Such as Ibogaine for Pharmacy in Japan

**DOI:** 10.7759/cureus.116178

**Published:** 2026-09-13

**Authors:** Robert B Raffa

**Affiliations:** 1 School of Pharmacy, Temple University, Philadelphia, USA

**Keywords:** drug interactions, drug regulation, ibogaine, japan pharmacy practice, pmda, psychedelics

## Abstract

An Executive Order signed in April 2026 directed US federal agencies to expedite development and review pathways for psychedelic and psychedelic-adjacent (such as ibogaine) therapies. The FDA issued Commissioner's National Priority Vouchers to psilocybin developers, and cleared the investigational new drug (IND) application for noribogaine hydrochloride for alcohol use disorder. These developments make FDA approval of at least one psychedelic or psychedelic-adjacent agent within a compressed timeframe a foreseeable regulatory event. The potential implications for pharmacy in Japan - a country with a highly structured drug classification and distribution system, culturally and legally embedded counseling obligations on pharmacists, and relatively conservative narcotics control legislation that directly schedules psilocybin, MDMA, LSD, and ibogaine - is an interesting question. This narrative review was prepared from published peer-reviewed literature in the English and Japanese languages identified through PubMed/MEDLINE, with supplemental journal searches, federal surveillance data, and published analyses of the Japanese Pharmaceuticals and Medical Devices Agency (PMDA). References were selected for relevance and recency (prioritizing 2020 - 2026). The US FDA approval of a psychedelic or psychedelic-adjacent agent would not trigger an automatic regulatory response in Japan, where all relevant compounds are already scheduled under the Narcotics and Psychotropics Control Act (2024 revision). However, three types of pressure would follow: regulatory pressure through the PMDA initiatives to reduce drug approval time; clinical pressure through Japanese psychiatrists and patients seeking novel therapies for treatment-resistant depression (TRD); and pressure on pharmacists because of legal requirements for drug interaction counseling, information on harm reduction options, and pharmacogenomic guidance. Ibogaine would present the most pharmacologically demanding scenario for Japanese pharmacy, requiring CYP2D6 metabolizer phenotype assessment (38-50% frequency of the reduced-function CYP2D6*10 allele in Japan), mandatory QT-prolonging drug interaction screening, and cardiac monitoring, all of which do not currently exist in community pharmacy settings. Over-the-counter (OTC) status for psychedelic and psychedelic-adjacent agents is legally, culturally, and regulatorily remote under current Japanese law, but the counseling role of the Japanese pharmacist (yakuzaishi, 薬剤師) positions them as critical and uniquely qualified resources regarding psychedelic and psychedelic-adjacent products and for the drug interaction challenges that approval of these therapies in other countries would create.

## Introduction and background

The regulation of psychoactive substances in any country involves overlapping aspects of pharmacology, pharmacy, public health policy, cultural values, and criminal law, and nowhere is the intersection more distinctively configured than in Japan [1]. As the United States moves toward possible - even likely - FDA approval of psychedelic-adjacent or psychedelic-adjacent therapies [2], Japanese pharmacy faces a set of questions, including: How will Japan's Pharmaceuticals and Medical Devices Agency (PMDA) respond to an FDA approval of psilocybin, MDMA, ibogaine, or similar compounds? What obligations will Japanese pharmacists carry for drug interaction counseling if psychedelic and psychedelic-adjacent products reach the market? Could any psychedelic or psychedelic-adjacent therapy ever achieve over-the-counter (OTC) status in Japan? And what does ibogaine's uniquely demanding pharmacologic and safety profile mean for a pharmacy system like Japan's, which is built around strict control and custom?

These questions are not hypothetical. An Executive Order signed by the United States President on April 18, 2026 [3], directed US federal agencies to expedite development and review of psychedelic and psychedelic-adjacent therapies for mental health conditions, resulting in FDA issuance of Commissioner's National Priority Vouchers to companies developing psilocybin and methylone, potentially compressing standard review timelines to only about 1-2 months [4]. At the same time, the FDA cleared the investigational new drug (IND) application for a clinical study of noribogaine hydrochloride, the primary active metabolite of ibogaine, for treatment of alcohol use disorder [5]. The FDA had already granted breakthrough therapy designation to psilocybin for treatment-resistant depression (TRD) [6]. These developments collectively portend a compressed approval timeline that could possibly prompt an international regulatory cascade of approval.

Japan's regulatory and cultural context differs from that of the United States in ways that are relevant to pharmacy. Psilocybin, MDMA, LSD, and ibogaine are scheduled as narcotics under Japan’s Narcotics and Psychotropics Control Act (麻薬及び向精神薬取締法), with less developed exception framework than the FDA's breakthrough therapy designation pathway [7]. The 2024 amendment to Japan's Cannabis Control Act, which criminalized cannabis use and moved THC under the Narcotics and Psychotropics Control Act, signals that Japan's legislative trajectory is currently moving toward stricter psychoactive substance control, albeit with therapeutic accommodation for certain substances [8]. Yet Japan also has a substantial unmet need in psychiatric pharmacotherapy. For example, treatment-resistant depression (TRD) affects an estimated 12% of the Japanese population, suggestive of a large population for whom conventional antidepressant pharmacotherapy has not been efficacious [9]. The intersection of a strict narcotics law, unmet mental-disorder need, and an imminent international approval event creates the regulatory and clinical context within which Japanese pharmacy could be faced.

## Review

The Japanese pharmacy (薬局) system and the Yakuzaishi's role

Under Japanese law and professional oversight by the Ministry of Health, Labour, and Welfare (MHLW), ‘pharmacies’ are facilities allowed to dispense prescription medications under strict regulatory and legal requirements. A ‘drugstore’ is a retail business (‘store sales business’) allowed to sell over-the-counter (OTC) pharmaceuticals and daily necessities, but not prescription medications. Most ‘drugstores’ do both, thus are more analogous to ‘retail’ or ‘community’ pharmacies in the US. It is also common to have a medication-dispensing location within a hospital, which is usually called a ‘pharmacy department’, ‘dispensing office’, ‘hospital pharmacy’, or just ‘pharmacy’. The certified and registered professional employed at any of these locations is called a ‘pharmacist’ because of their professional pharmacy-school degree, independent of site of practice. Japan’s OTC businesses are also strictly controlled, but do not dispense prescription medications. Therefore, the concentration in the present communication is on dispensing pharmacies, but it is helpful to include reference to OTC regulations in order to explain why a ‘switch’ of a psychedelic or psychedelic-adjacent drug to OTC status currently would be very unlikely.

**Figure 1:**
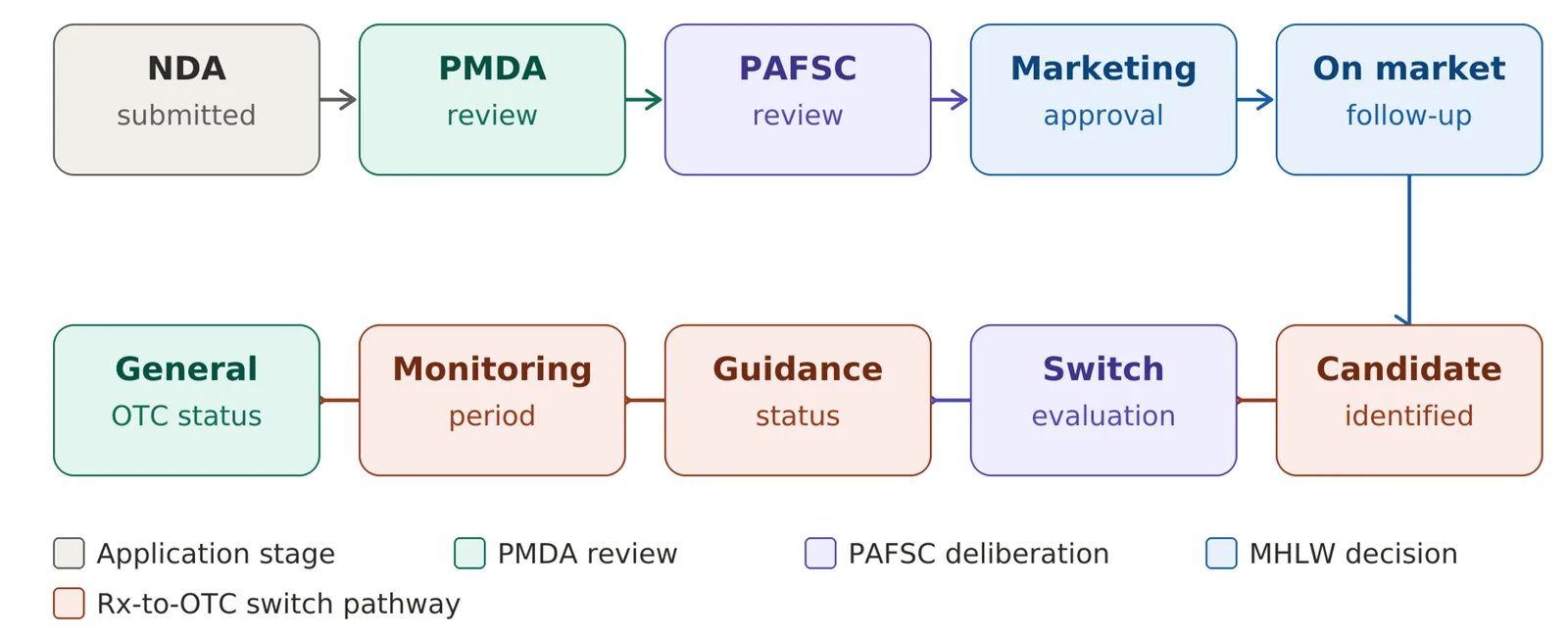
A generalized life-cycle of a drug in Japan, including OTC status if possible NDA (also J-NDA): new drug application; PMDA: Pharmaceuticals and Medical Devices Agency; PAFSC: Pharmaceutical Affairs and Food Sanitation Council; Rx: prescription; OTC: over-the-counter.

Japan's medication market is governed by the recent 2025 edition of the Pharmaceutical and Medical Devices Act (PMD Act, 薬機法), which divides products into a hierarchy of risk categories, and also defines the pharmacist's legal obligations related to each category [5,6]. Understanding this system is a prerequisite to analyzing how a psychedelic or psychedelic-adjacent product would fit within it, because the classification determines not merely labeling requirements, but also the specific legal duties of the yakuzaishi at point of sale. A part of Japan’s regulations is the category of Guidance-Requiring Pharmaceuticals (要指導医薬品, yō-shidō iyakuhin), introduced by the 2013 PMD Act amendment, which encompasses ‘switch-OTC’ drugs (スイッチOTC) that have recently transitioned from prescription status, and direct-OTC drugs newly entering the market without prior prescription history (Figure 1) [10,11].

Sale of the products is subject to stringent requirements under PMD Act Article 36 [12]: requiring face-to-face sales only; pharmacist-only sales; mandatory pharmacist confirmation of purchaser identity, age, concurrent medications, allergies, and intended use before sale; comprehensive information and guidance provision using pharmaceutical expertise; and quantity restrictions limiting sale to the minimum necessary for one person's use. This category represents a generally higher standard of pharmacist gatekeeping than currently applied in American community pharmacies. Below the top category is Class 1 OTC (第一類医薬品), which covers medications with potential for serious side effects sufficient to interfere with daily life, including H2 receptor antagonists and high-dose minoxidil, which require mandatory pharmacist information provision before sale and face-to-face interaction. Class 2 OTC (第二類医薬品), which covers common cold remedies, antipyretics, and analgesics, require pharmacist or registered seller counseling on a best-effort basis. Class 3 OTC (第三類医薬品) covers vitamins and intestinal preparations without mandatory counseling.

**Figure 2:**
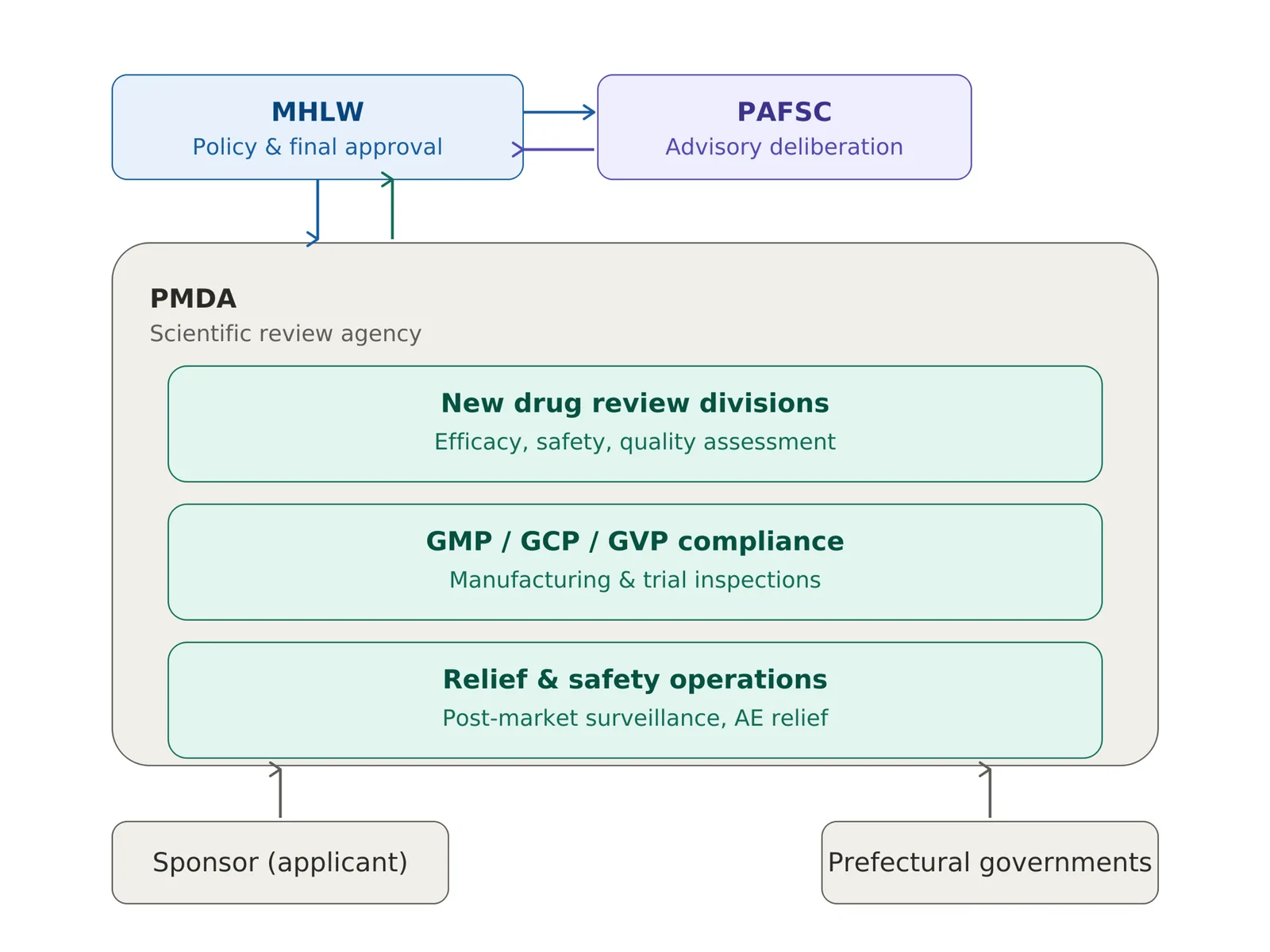
Tiered structure for regulatory drug control in Japan PAFSC: Pharmaceutical Affairs and Food Sanitation Council; MHLW: Ministry of Health, Labour, and Welfare; PMDA: Pharmaceuticals and Medical Devices Agency.

This tiered structure means that a psychedelic or psychedelic-adjacent product entering the Japanese market would almost certainly be classified as a Guidance-Requiring Pharmaceutical, and no lower than a Class 1 OTC, the two categories that require mandatory, face-to-face counseling by a pharmacist (Figure 2).

The yakuzaishi's legal obligation for medications in these categories extends beyond just product description. It includes active assessment of the user's health status, medication appropriateness, concurrent medications, and guidance for use. This pre-existing infrastructure positions the Japanese pharmacy as a structurally more stringent gatekeeper for high-risk CNS-active products than the US community pharmacy model, and is contingent on yakuzaishi receiving appropriate training in psychedelic and psychedelic-adjacent pharmacology and drug interactions.

Japan's regulatory framework for psychedelics and psychedelic-adjacents

In Japan, all classical psychedelic and psychedelic-adjacent substances are currently scheduled as narcotics (麻薬, mayaku) under the Narcotics and Psychotropics Control Act, consistent with Japan's obligations under the United Nations Convention on Psychotropic Substances of 1971, under which LSD, MDMA, and psilocybin are ‘Table I’ substances, and ibogaine appears in some international scheduling discussions [8]. This scheduling status creates the highest tier of legal restriction: manufacture, possession, import, export, and administration require specific licenses issued by the governors of each Prefecture, and therapeutic use without formal approval and rescheduling is legally prohibited. Japan's 2024 tightening of cannabis regulations, moving in the opposite direction from global trends toward decriminalization, illustrates that domestic political and cultural norms trend to restrictive frameworks [8].

Japan has historically experienced significant drug-lag, i.e., the interval between first global approval and PMDA approval, particularly for novel psychiatric agents. For example, among 400 new drugs approved in Japan between 2008 and 2019, 80% were first approved outside Japan, with 50.5% of cases first approved in the US [7]. However, the median drug-lag has declined substantially, from over 600 days in 2005 to approximately 330 days in 2023, driven by initiatives including the SAKIGAKE designation system (introduced in 2015 for pioneering drugs addressing unmet needs), the conditional early approval system, and the December 2023 MHLW guideline, waiving the mandatory Japanese Phase I study for drugs supported by comparable foreign data [13]. Nevertheless, psychedelic and psychedelic-adjacent drugs present a challenge to even the expedited PMDA pathway: SAKIGAKE designation requires that a product be first developed in Japan, a condition that US-approved drugs would not meet, and the rescheduling of a narcotic under the Narcotics and Psychotropics Control Act requires a separate legislative or ministerial process independent of the PMDA drug approval pathway [14]. Esketamine (Spravato) offers a useful precedent: approved by the FDA in March 2019, it received PMDA approval in Japan in September 2021, a lag of approximately 30 months [13]. Esketamine is a dissociative anesthetic with controlled substance implications, yet it navigated the PMDA pathway in a reasonable timeframe. Psilocybin, if FDA-approved, would face the additional hurdle of rescheduling from narcotic designation before clinical use could proceed, a step with no fixed timeline in Japanese regulatory procedures.

The pathway most likely to accelerate Japanese psychedelic and psychedelic-adjacent approval is the 2022 Emergency Approval system, which was enacted as a formal PMD Act amendment inspired by the COVID-19 experience, and allows time-limited approval based on “presumed efficacy” during public health emergencies [8]. Japan's mental health crisis, exacerbated by the COVID-19 pandemic, could theoretically provide the public health emergency justification needed to invoke this pathway, though its application to a psychedelic or psychedelic-adjacent agent with controlled substance implications would be an unprecedented use of this mechanism.

The unmet clinical need of a mental health burden in Japan

The clinical pressure that might drive demand for psychedelic and psychedelic-adjacent therapies in Japan is substantial and well-documented. For example, a retrospective claims database study, the first to provide population-based estimates for TRD in Japan, found a real-world TRD prevalence of approximately 12% among patients receiving pharmacotherapy for depression [9]. Post-COVID-19 data from a national survey of 1,765,102 participants found that the prevalence of more severe psychological distress increased between 2019 and 2022, with the largest increases concentrated among working-age adults aged 35-49 years [15]. The World Mental Health Japan Survey documented that a substantial proportion of patients with severe mental disorders receive no treatment, with substance use disorders showing particularly low treatment rates, reflecting both stigma and resource constraints in the Japanese mental health system [16].

For Japanese psychiatrists and their patients, the international evidence base for psilocybin-assisted therapy in TRD, including the landmark Phase 3 COMP360 trial demonstrating apparent antidepressant effects from a single 25 mg dose [17], will be accessible through the same scientific literature that informs clinical practice globally. The result will be a growing cohort of Japanese patients with TRD who become aware of a therapy available in the United States that may produce durable remission, whereas four or more ‘conventional’ antidepressant trials have failed. This awareness could prompt demand for ‘medical tourism’ to obtain psychedelic or psychedelic-adjacent therapy, and requirements for Japanese pharmacists to counsel these patients, in addition to potential abuse concerns, about potential drug interactions between their existing psychiatric medications and any psychedelic or psychedelic-adjacent agents they may have obtained abroad or may intend to seek. Thus, the counseling obligation for Japanese yakuzaishi could arise from clinical reality before it arises from regulatory authorization.

Drug interaction and yakuzaishi: what Japanese pharmacists need to know

The most likely clinically prevalent drug interaction concern for Japanese dispensing pharmacists counseling patients about psychedelic and psychedelic-adjacent therapies involves combinations of serotonergic agents. Selective serotonin reuptake inhibitors (SSRIs) and serotonin-norepinephrine reuptake inhibitors (SNRIs) are widely prescribed for depression and anxiety in Japan. Chronic SSRI/SNRI use attenuates the subjective and therapeutic effects of serotonergic psychedelics, particularly psilocybin and LSD, through 5-HT2A receptor downregulation, with the blunting effect persisting for up to three months after SSRI discontinuation [18]. A scoping review of concomitant antidepressant and psychedelic-adjacent use suggested that while clinically documented serotonin syndrome during supervised SSRI-psilocybin co-administration is rare, the MAOI (monoamine oxidase inhibition) content of ayahuasca (psychedelic-adjacent decoction from botanical sources) renders its combination with a serotonergic agent a genuine concern [18].

Lithium, widely used in Japan for bipolar disorder and augmentation of antidepressant therapy, is associated with seizures in approximately 47% of documented cases when combined with classical serotonergic psychedelics, one of the most serious drug-interaction contraindications in psychedelic medicine [19]. The yakuzaishi's legal obligation to confirm concurrent medications before dispensing any Guidance-Requiring or Class 1 OTC product directly encompasses this interaction. However, acting on it requires familiarity with psychedelic and psychedelic-adjacent pharmacology that has, to date, been generally minimal in Japanese pharmacy curriculum.

Although OTC pharmacists would unlikely ever dispense psychedelics or psychedelic-adjacent drugs, a uniquely Japanese pharmacological drug-interaction concern involves Kampo (漢方薬) preparations - traditional herbal medicines that are classified as approved drugs in Japan and sold OTC in drugstores (yakkyoku) across the country. An estimated 80% of Japanese physicians prescribe Kampo medicines, and many Kampo products are available without prescription [11]. Of particular relevance to psychedelic and psychedelic-adjacent drug interactions are Kampo preparations containing St. John's Wort (Hypericum perforatum), known in Japan as seiryu preparations, which is sold OTC for mild depression and is a potent inducer of CYP3A4 - St. John's Wort's CYP3A4 induction would be expected to reduce plasma concentrations of CYP3A4-metabolized psychedelics and psychedelic-adjacents [20]. The pharmacist who sells Kampo preparations will be required to have counseling competency about the various types of drug interactions.

Several additional product classes commonly used in Japan have interaction concerns regarding specific psychedelics or psychedelic-adjacents. Antihistamines, which are widely used OTC for allergy and as sleep aids in Japan, include first-generation agents (e.g., diphenhydramine, chlorpheniramine) that have anticholinergic and CNS depressant properties. Japanese OTC analgesics containing codeine remain available in some formulations under controlled sales conditions; yakuzaishi who counsel patients about CYP2D6-metabolized drugs should recognize that many CNS drugs share the CYP2D6 pathway of ibogaine metabolism [21]. Likewise, caffeine-containing OTC preparations, ubiquitous in Japanese drugstores, are metabolized by CYP1A2 [22]. While this specific interaction is unlikely to be clinically significant at standard caffeine doses, it illustrates the broad extent of CYP1A2-relevant considerations that psychedelic and psychedelic-adjacent therapy would create for counseling by a pharmacist.

Ibogaine: a pharmacologically demanding scenario for Japanese pharmacy

Ibogaine, a Schedule I indole alkaloid under US law and a narcotic under Japan's Narcotics and Psychotropics Control Act, presents the most pharmacologically complex scenario of any agent currently in the psychedelic and psychedelic-adjacent development pipeline. Its multi-receptor pharmacology, involving NMDA glutamate receptors, nicotinic acetylcholine receptors, mu- and kappa-opioid receptors, and monoamine transporters, underlies its complex pharmacology and safety profile [23]. In the Japanese context, the substance use disorder treatment application most likely to drive clinical interest in ibogaine is not opioid use disorder, which is substantially less prevalent in Japan than in the United States, but methamphetamine use disorder, for which Japan has a higher prevalence, and for which ibogaine has shown preliminary anti-CNS-stimulant effects in preclinical models [24].

Ibogaine's metabolic clearance is via CYP2D6 activity. A clinical pharmacokinetic-pharmacodynamic study demonstrated that ibogaine clearance at a CYP2D6 activity score of zero is 0.82 L/h, increasing by 30.7 L/h for each unit of CYP2D6 activity score [21]. This pharmacogenomic dependency has specific and important implications in Japan, where the CYP2D6 allele frequency distribution differs substantially from Western populations. The CYP2D6*10 allele, a reduced-function variant encoding an enzyme with markedly lower activity than wild-type, reaches allele frequencies of 38-50% in Japanese and broader East Asian populations, compared to approximately 1-2% in Europeans [25]. Such patients would accumulate higher plasma concentrations of ibogaine than would more rapid metabolizers at equivalent doses, amplifying cardiac risk. The cardiac safety profile of ibogaine, which inhibits the hERG potassium channel and Nav1.5 sodium channel, producing QTc prolongation as a substrate for torsades de pointes and potentially fatal ventricular arrhythmia, is directly amplified in CYP2D6 poor or intermediate metabolizers [23,26]. A systematic review of ibogaine-related fatalities found that unsupervised use, concurrent QT-prolonging medications, electrolyte abnormalities, and CYP2D6 inhibitor co-administration were the most consistently identified risk factors [26]. Not all Japanese pharmacists currently have the infrastructure for mandatory pre-dispensing CYP2D6 genotyping, ECG assessment, Mg^2+^, or electrolyte monitoring, the clinical prerequisites established as necessary for safe administration of ibogaine [21]. Any path to ibogaine's clinical approval in Japan would require the development of this infrastructure. The systematic pre-administration screening for QT-prolonging drug co-medications identified as essential to ibogaine safety [26] requires the yakuzaishi to review a medication list spanning virtually all major drug classes. QT-prolonging agents common in the Japanese prescription list include: antipsychotics widely prescribed for schizophrenia and depression augmentation (e.g., haloperidol, quetiapine, and sulpiride, the latter used more commonly in Japan than in Western practice for functional gastrointestinal disorders); fluoroquinolone antibiotics widely used in primary care; macrolide antibiotics; azole antifungals; antiemetics such as domperidone; and, most critically for the abuse treatment context, methadone, though methadone's role in Japanese opioid use disorder treatment is more limited than in the US [26]. Sulpiride's widespread Japanese OTC and prescription use for gastrointestinal indications without explicit cardiac monitoring, a distinctly Japanese prescribing pattern, is a specific and underappreciated QT interaction concern in the ibogaine context.

OTC status for psychedelic and psychedelic-adjacent products

OTC ibogaine in Japan anytime soon is unlikely. A drug requiring pre-administration CYP2D6 genotyping, 12-lead ECG assessment, electrolyte measurement, Mg2+, and continuous cardiac monitoring during administration cannot be safely dispensed OTC under any drug classification framework. The Japanese OTC system's most stringent category, Guidance-Requiring Pharmaceuticals, mandates face-to-face pharmacist counseling and prohibits internet sales, but it does not create the infrastructure for pre-dispensing genetic testing and ECG monitoring. Thus, ibogaine OTC status in Japan is not a near-term plausible scenario under a foreseeable regulatory model.

If psilocybin or MDMA receive FDA approval, the most likely US regulatory model is a Risk Evaluation and Mitigation Strategy (REMS) requiring certified healthcare settings, trained providers, and specialty pharmacy dispensing [27]. The Japanese equivalent of a REMS-constrained dispensing model would be a prescription drug designation (医療用医薬品) with specific institutional use restrictions, analogous to the model used for clozapine (クロザリル), which requires mandatory absolute neutrophil count monitoring before each dispensing event and can only be dispensed by registered hospital pharmacies. Psilocybin and MDMA-like therapies would, if ever approved in Japan, almost certainly follow a similar path: hospital-based or registered clinic dispensing, not drugstore or OTC settings. The yakuzaishi's role in this model would be analogous to the hospital pharmacist's role in clozapine dispensing, i.e., verification of safety criteria, documentation, and provider communication, rather than a retail counseling encounter.

The most plausible scenario for psychedelic or psychedelic-adjacent products approaching OTC availability in Japan in the near term involves harm reduction rather than therapeutic administration. As substance use increases among younger Japanese adults [28], demand for reagent testing kits (for identifying adulterants in street substances) and drug interaction information resources may emerge. These products are not drugs under the PMD Act and would be regulated as quasi-drugs or consumer products rather than OTC pharmaceuticals, potentially reducing the regulatory barrier to availability. The pharmacist's counseling role in this context would be to provide scientifically accurate harm reduction information, a role that requires the pharmacology competency regardless of the product's classification.

‘Sub-perceptual’ microdosing of psilocybin (0.1-0.3 g) has attracted scientific and popular interest for mood and cognitive applications [29,30], and there is even early-stage international regulatory discussion about whether well-characterized low-dose psilocybin formulations with a defined safety profile could eventually approach the status of approval. In Japan, this scenario would require not only demonstrated clinical safety at microdose levels, but perhaps rescheduling of psilocybin from narcotic status, which is a legislative process with no fixed timeline and no current political momentum in a country that in 2024 moved to criminalize non-medical cannabis use. Nevertheless, Japan's drug evaluation framework does include a formal reclassification process through the MHLW, including a mechanism for public and stakeholder input, introduced in the 2015 reform [11]. A microdosing psilocybin application through this process is technically possible in legal-structural terms, but would likely face opposition from medical professional associations, regulatory conservatism in CNS drug reclassification, and the prevailing political environment around psychoactive substances. The yakuzaishi community's own professional stance on such a reclassification, currently untested, could be a significant determinant of outcome.

The yakuzaishi as gatekeeper

Across all scenarios analyzed above, from REMS-equivalent prescription dispensing to harm reduction counseling to interaction screening for patients who have undergone psychedelic-adjacent therapy abroad, the Japanese pharmacist's existing legal framework and professional infrastructure position the yakuzaishi as a uniquely capable gatekeeper [31,32]. The PMD Act's mandatory face-to-face counseling requirements encode a standard of pharmacist engagement that exceeds what is legally required in most US pharmacy settings. The yakuzaishi's obligation to confirm concurrent medications, assess appropriateness of use, and provide comprehensive pharmaceutical guidance is precisely the clinical workflow required for safe psychedelic-adjacent drug interaction screening.

Japanese pharmacy education, governed by the six-year pharmacy degree program (薬学部) mandated since 2006 [33], includes comprehensive instruction in pharmacology, drug interactions, and CYP enzyme pharmacogenomics. CYP2D6 pharmacogenomics is taught in the context of codeine, tricyclic antidepressants, and beta-blockers, with specific attention to the CYP2D6*10 allele frequency in East Asian populations [20]. This existing pharmacogenomics curriculum provides a foundation for ibogaine-specific pharmacogenomic counseling that requires extension, but not reconstruction. Similarly, the serotonin syndrome interaction framework taught in the context of SSRIs and monoamine oxidase inhibitors, which directly applies to the psychedelic and psychedelic-adjacent drug interaction landscape, would require supplementation with drug-specific data rather than a fundamentally new conceptual framework.

Professional pharmacy organizations in Japan, including the Japanese Pharmaceutical Association (日本薬剤師会), will need to develop specific continuing education programs in psychedelic and psychedelic-adjacent pharmacology, ibogaine cardiac safety, CYP1A2/CYP2D6 interaction profiles relevant to these agents, and Kampo interactions [34], before the regulatory approval of these agents creates an immediate and overwhelming practice demand. The precedent of emergency contraception, which received nationwide OTC approval in Japan in October 2025 after a pilot program launched in November 2023, with mandatory pharmacist face-to-face counseling as a condition of sale [35], illustrates that Japanese pharmacies can adapt rapidly to high-stakes new products when the educational and institutional foundation is prepared in advance.

## Conclusions

US FDA approval of a psychedelic or psychedelic-adjacent agent, which is a regulatory event that has moved from speculative to plausible within a timeframe of months following the April 2026 Executive Order and FDA priority review designations, will unlikely trigger automatic regulatory approval in Japan, where psilocybin, MDMA, and ibogaine remain scheduled as narcotics under the Narcotics and Psychotropics Control Act. The historical PMDA drug-lag for novel psychiatric agents, averaging approximately 330 days in recent years, but extending to 30 months for esketamine, and the additional barrier of narcotic rescheduling as a prerequisite to PMDA approval, means that the interval between US approval and Japanese clinical availability will likely be measured in years. However, the implications of US psychedelic and psychedelic-adjacent approval for Japanese yakuzaishi will not wait for PMDA authorization. Japanese patients with TRD, approximately 12% of those receiving antidepressant pharmacotherapy, will access the international evidence base and pursue psychedelic or psychedelic-adjacent therapy through medical tourism or other channels. They will return to Japanese pharmacists with questions about drug interactions between their psychiatric medications, Kampo preparations, and the psychedelic or psychedelic-adjacent agent they have received. The yakuzaishi's legal obligation to provide comprehensive pharmaceutical guidance in face-to-face interactions is precisely the infrastructure needed to manage these interactions safely, provided the professional knowledge base is in place.

Ibogaine requires specific and urgent attention in Japanese pharmacy education for two reasons unique to the Japanese pharmacogenomic context. First, the CYP2D6*10 allele reaches 38-50% frequency in the Japanese population, producing a higher prevalence of intermediate metabolizer phenotype than in Western populations and systematically amplifying ibogaine's cardiac risk through reduced drug clearance. Second, sulpiride, a benzamide antipsychotic used widely in Japan for both psychiatric and gastrointestinal indications, has a QT-prolonging profile, thus represents a uniquely Japanese drug interaction concern for ibogaine that Western use would not. Rapid approval of ibogaine in Japan is currently highly unlikely under a foreseeable regulatory framework; its cardiac and pharmacogenomic requirements demand a level of pre-administration clinical assessment that exceeds the infrastructure of many pharmacy settings.

The yakuzaishi, equipped with Japan's most rigorous counseling obligations and a pharmacogenomics curriculum that already addresses CYP2D6 East Asian allele frequencies, is structurally well positioned to serve as a safe gatekeeper for psychedelic and psychedelic-adjacent therapies, that is, if the profession proactively develops the specific competencies that this therapeutic frontier requires.
